# Acceptability and feasibility of a mobile health application for enhancing public private mix for TB care among healthcare Workers in Southwestern Uganda

**DOI:** 10.1186/s44247-023-00009-0

**Published:** 2023-03-03

**Authors:** Wilson Tumuhimbise, Daniel Atwine, Fred Kaggwa, Angella Musiimenta

**Affiliations:** 1 Mbarara University of Science and Technology, Faculty of Computing and Informatics, P.O. Box 1410, Mbarara, Uganda; 2 SOAR Research Foundation, Mbarara, Uganda; 3 Angels Compassion Research and Development Initiative, Mbarara, Uganda

**Keywords:** Acceptability, Feasibility, Mobile application, Public–private mix, Tuberculosis

## Abstract

**Background:**

Mobile health interventions can potentially enhance public–private linkage for tuberculosis care. However, evidence about their acceptability and feasibility is lacking. This study sought to assess the initial acceptability and feasibility of a mobile health application for following up on presumptive tuberculosis patients referred from private to public hospitals. Twenty-two healthcare workers from three private hospitals and a public hospital in southwestern Uganda received the Tuuka mobile application for 1 month for testing. Testing focused on referring patients by healthcare workers from private hospitals and receiving referred patients by public healthcare workers and sending SMS reminders to the referred patients by filling out the digital referral forms inbuilt within the app. Study participants participated in qualitative semi-structured in-depth interviews on the acceptability and feasibility of this app. An inductive, content analytic approach, framed by the unified theory of acceptance and use of technology model, was used to analyze qualitative data. Quantitative feasibility metrics and the quantitative assessment of acceptability were analyzed descriptively using STATA.

**Results:**

Healthcare workers found the Tuuka application acceptable and feasible, with a mean total system usability scale score of 98 (SD 1.97). The majority believed that the app would help them make quicker medical decisions (91%), communicate with other healthcare workers (96%), facilitate partnerships with other hospitals (100%), and enhance quick TB case notification (96%). The application was perceived to be useful in reminding referred patients to adhere to referral appointments, notifying public hospital healthcare workers about the incoming referred patients, facilitating communication across facilities, and enhancing patient-based care.

**Conclusion:**

The Tuuka mobile health application is acceptable and feasible for following up on referred presumptive tuberculosis patients referred from private to public hospitals in southwestern Uganda. Future efforts should focus on incorporating incentives to motivate and enable sustained use among healthcare workers.

**Supplementary Information:**

The online version contains supplementary material available at 10.1186/s44247-023-00009-0.

## Introduction

Tuberculosis (TB) is ranked as the 13th leading cause of death globally and the second leading infectious disease after coronavirus disease (COVID-19) [1], despite being curable and preventable. A total of 10 million people fell ill with TB, while 1.5 million deaths were registered globally in 2020. The majority (98%) of these reported cases are in low- and middle-income countries; for example, Africa has the highest TB incidence: 220 (195–245) per 100,000 compared to the rest of the world [1]. In Uganda, the TB incidence is 196 (117–296) per 100,000 and 65 (39–98) per 100,000 among people living with HIV, making it among the 30 high TB burden countries and 30 high TB/HIV burden countries [2]. Although efforts to control TB care are underway, the public hospitals that are at the forefront of TB care service provision are overwhelmed with a high number of patients.

In an effort to enhance the commitment to eliminate TB globally, the involvement of private healthcare facilities has been recommended by the World Health Organization (WHO) as a key subcomponent of the End TB strategy pillar *“bold policies and supportive systems*” [3]. In 2003, the WHO birthed the Public Private Mix (PPM) in an effort to involve all healthcare providers in the provision of TB care services [4]. PPM involves several strategies and mechanisms that link health entities from both private and public facilities to the National Tuberculosis and Leprosy programs for the expansion of Directly Observed Treatment Short course (DOTS) activities [1]. PPM implementation globally has resulted in improved notification and retention of TB cases in Nigeria and India, respectively [5, 6], and reduced financial burden on patients receiving TB care in the private sector [7].

Private hospitals are perceived to be near or within close proximity to patients and to provide high quality of care to patients by ensuring privacy and confidentiality. However, concerns regarding lack of accreditation and proper follow-up mechanisms hinder their engagement in TB care [8]. The majority of these private hospitals refer presumptive TB patients to government hospitals for better TB management. However, the success of this referral process is not guaranteed since the majority of private hospitals lack proper follow-up mechanisms for ensuring that TB patients reach the facilities where they are referred [8]. Patients often delay going to referral units due to reluctance to transfer from private to public facilities, with some ending up being lost to follow-up on referral [9, 10]. This has led to increased delayed diagnosis, treatment initiation and consequent increased disease severity, prolonged duration of infectivity and transmission at the household level. This results in catastrophic costs for patients and their families [11], increases the risk of drug resistance [12], worsens the patients’ health conditions, and eventually leads to death in the long run [13].

The utilization of mobile health technologies has been found to be feasible and acceptable in low-resource settings [14, 15] and has shown potential to reduce the amount of time spent at health facilities [16], enhance patients’ medication adherence [17, 18] and enhance the involvement of the private sector in TB care [19]. However, literature regarding their acceptability and feasibility in enhancing PPM, especially in African settings, remains limited. Understanding the acceptability and feasibility of mobile health technologies is important in laying the foundation for the usability, adoption, and utilization of such technologies. Given the promise of mobile health interventions in supporting TB care, studies about how acceptable and feasible these technologies are, especially in enhancing PPM, are needed. Several studies have been carried out to assess the feasibility and acceptability of these mobile health interventions elsewhere. For example, in Indonesia, Lestari and colleagues found a referral and reporting back mobile smartphone application feasible [20]. In British Columbia, a pilot intervention study implementing the *Weltel* intervention utilizing automated SMS messages from a computer system at a clinic was found to be feasible [21]. However, evidence on the feasibility and acceptability of mobile health interventions for following up on referred presumptive TB patients from private to public hospitals in Uganda is lacking. This paper addresses this gap by exploring the initial acceptability and feasibility of the Tuuka mobile application for following up presumptive TB patients referred from private to public hospitals in southwestern Uganda. The Tuuka mobile application is built on a centralized patient management scenario to provide patient-centered TB care that involves bringing together the parties involved in the patient’s pathway to effectively manage the patient, where private hospitals notify public hospitals about the referred presumptive TB patient.

## Methodology

### Study setting and participants

The methodology for this study has been described previously [8, 22]. Briefly, the participants were recruited from three private for-profit hospitals in Mbarara city in southwestern Uganda. These facilities were purposively selected for having relatively large medical establishments with a large number of healthcare workers, being involved in TB case detection, and being at the center of handling a large number of outpatients in Mbarara city, which increases the likelihood of detection of presumptive TB cases. After performing symptom-based screening of patients for TB, all hospitals refer the presumptive TB patients to Mbarara Regional Referral Hospital (MRRH). The investigation involved in-depth interviews with purposefully selected healthcare workers from the study sites to understand how feasible and acceptable an mHealth intervention for enhancing the public–private mix for TB care is. Feasibility was assessed in terms of having access to internet, compatible phones and access to electricity for charging these phones. Healthcare workers were trained on how to use the application and were able to use it. All healthcare workers owned smartphones, were able to use the application for a period of 1 month (February 2022) and were asked to participate in phone-based interviews.

### Study procedures

#### mHealth application (Tuuka app)

**Fig. 1:**
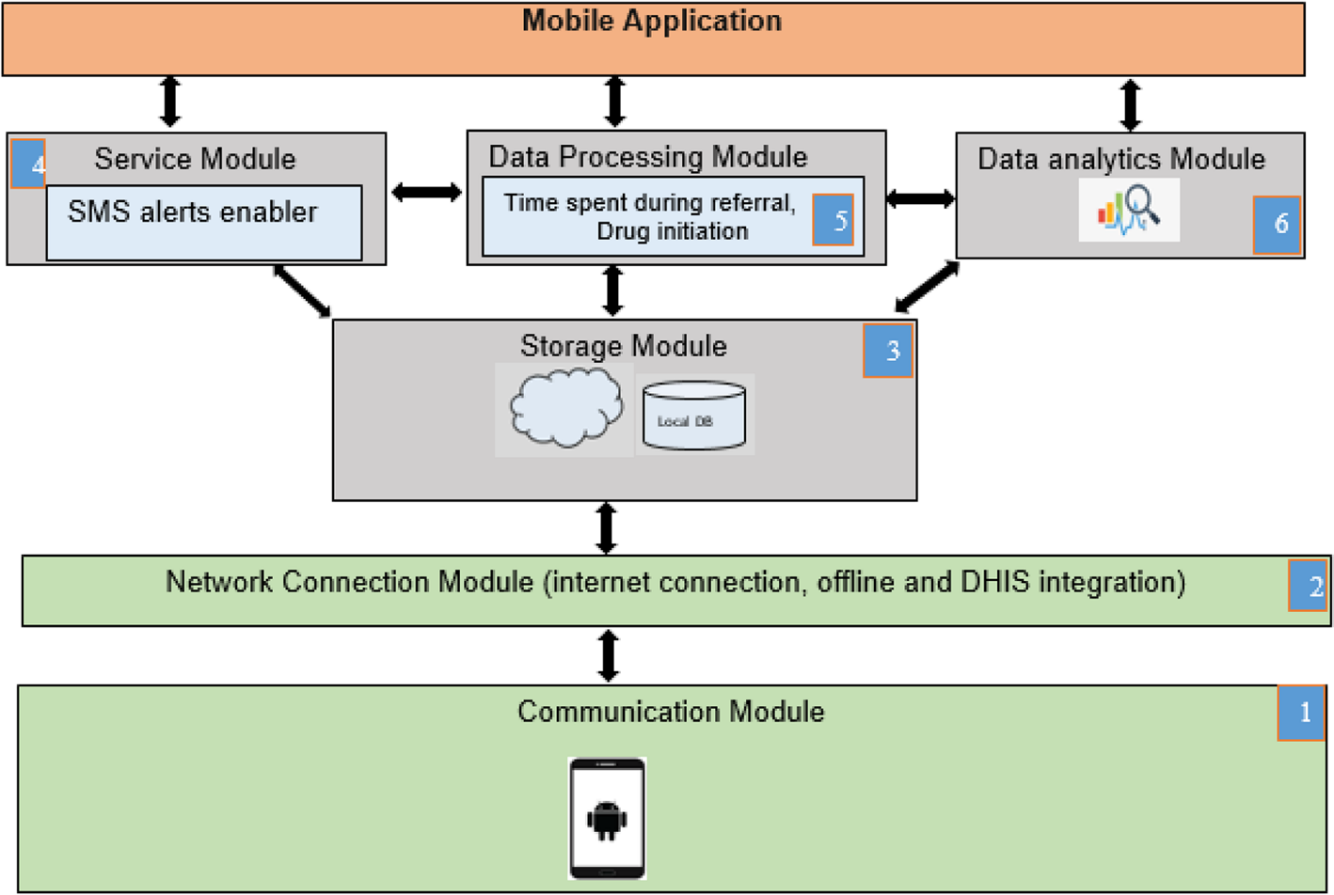
MOHE framework modules

The Tuuka application was developed based on the mobile health (MOHE) framework, composed of six modules responsible for the functionality of the developed application, as shown in Fig. 1 below.

1. Application layer module that offers an interface for an application to run on the Android platform
2. Service module responsible for sending and receiving short message service reminders to presumptive tuberculosis patients and healthcare workers
3. Network connection module responsible for the connectivity of the developed application to ensure that it functions
4. Storage module responsible for storage, access and retrieval of the patients’ data
5. Data processing module responsible for computing the time spent along the referral pathway
6. Data analytic module that enables the analysis and generation of reports from data collected on patients.

**Fig. 2:**
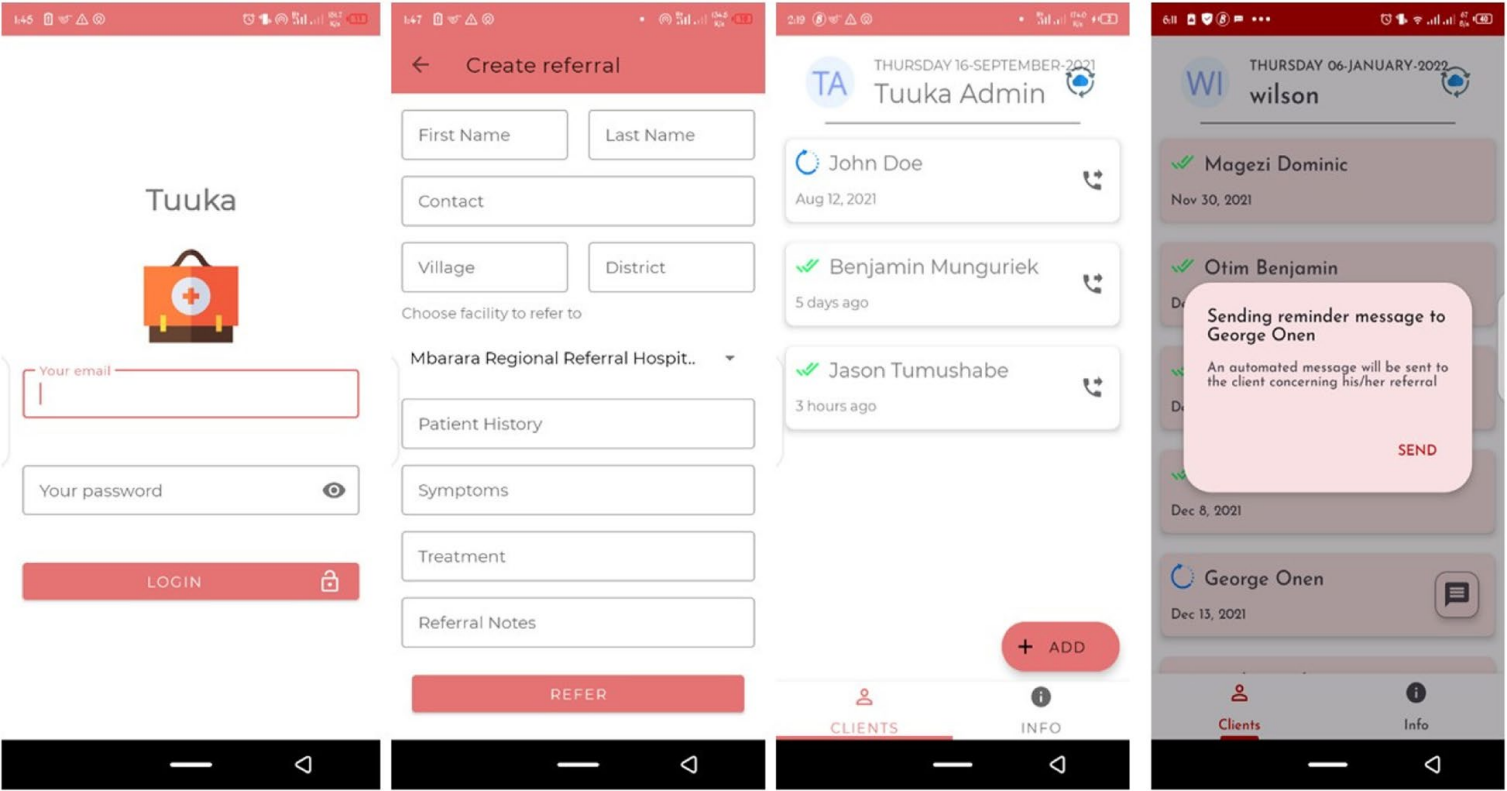
Screenshots from the Tuuka app

The application, as shown in Fig. 2 below, was developed following a user-centered design approach that involved input from the users (healthcare workers) during the development phase to understand the end user’s perspectives about the use of the app. Subsequent feedback was obtained from healthcare workers, and refinements and redesigning of the application was carried out until the desired prototype was reached. The word Tuuka is a Luganda word meaning “reach” and was chosen to represent the referral context in which the application was implemented. The application was deployed on healthcare workers’ compatible low-cost smartphones from three private hospital facilities who referred patients to a public hospital MRRH TB clinic to enable the notification of the referred presumptive TB cases.

#### Tuuka application implementation

Tuuka is made up of two main components, the application server, which is responsible for providing services to the users [23], and the client-side, which provides an interface between the services provided by the system and the users. The application server is the back end of the developed application, which responds to the requests and processes the received data and user authentications. The server is hosted by Linode with a cloud-based database for the storage of data. The application client is the front end that allows users (healthcare workers and system administrators) to interact with the system. The application can be accessed through both the web interface (for account creation and report generation by system managers) and the application interface for healthcare workers. Both the application and the web interface were developed using the Kotlin programming language. The application is available on the Google Play store https://play.google.com/store/apps/details?id=ug.global.mohe and the web application on https://tuuka.tech/.

The main users of the Tuuka application are healthcare workers, and its main functionality is capturing the referred patients’ data using built-in digital referral forms. The forms are designed to collect the same information collected through standard paper-based referral notes currently used by healthcare workers at private health facilities. Below are the procedures of Tuuka usability after application installation.

- The private healthcare worker enters the patients’ details, including names, telephone contacts, village, district, patient history, symptoms, treatment, and referral notes.
- The application automatically sends an SMS message to the patient’s mobile phone about the facility where they have been referred and an SMS notification to a healthcare worker at the place of referral about the incoming patient. If affirmative, the patient goes to the referral unit. The application automatically records the exact time and date the patient was referred and seen at the public hospital to understand whether the patient is affirmative or not to the referral process.
- Upon receiving an SMS notification about the incoming patient and receiving the patient at the facility, the public healthcare worker who already has an application installed notifies the private healthcare workers by saving the patients details and submitting the steps taken to handle the patient in terms of diagnosis and initiation of treatment.
- If a patient is not affirmative 12 hours after the time of referral, the public health worker notifies him or her about going to the referral unit through an automatic SMS message.

#### Authentication and security

Both mobile and web applications perform full encryption of databases, authentication and authorization. Users are required to authenticate themselves to access the information specific to them. Accounts are only created by the system administrator. The domain or connection to the server is through the Hypertext Transfer Protocol secure (HTTPS), which ensures that all communications are encrypted and Transport Layer Security (TLS) enforced at all times.

#### Data collection

All study participants received the final version of the application from the Google Play store and were trained by the principal investigator (WT), who explained to the healthcare workers and demonstrated how to use the application (e.g., logging in, adding the referral in the app). The healthcare workers were then asked to explain what the app does and to demonstrate practically how it operates. Of the 24 participants who received the application, two reported failure to download the application from the shared link and were not available to participate in data collection. Twenty-two participants who installed the application were contacted for a phone interview and the interviewer-administered questionnaire (Additional file 1) to assess their overall experience with using the intervention. Acceptability and feasibility were determined quantitatively using the system usability scale (SUS) [24]. The application would be considered acceptable if 90% of healthcare workers rate 70% (7/10) items on the SUS scale as “very good” or higher. Qualitatively, acceptability was assessed following the Unified Theory of Acceptance and Use of Technology (UTAUT) model [25]. UTAUT was used because it has been found to be a useful tool for assessing the likelihood of success for new technology and helps in understanding the factors for acceptance for proper design of interventions [25]. Semi-structured interviews were carried out among 18 private healthcare workers and four public healthcare workers until thematic saturation was reached (at the 22nd participant). All questions in the interview guide (Additional file 2) were pilot tested and guided and were based on the four main constructs of the UTAUT model. The questions elicited information regarding the study participants’ experiences of using the application, the ease of use, challenges encountered and the reaction by peers while using the application to document the acceptability and feasibility of the application. The data captured the perceived usefulness (performance expectancy) of the app, perceived ease of use (effort expectancy), social influence, and facilitating conditions. Each interview lasted between 30 and 45 minutes. A short survey was then administered to the participants to gather their social demographic details. In addition, a technology adoption survey based on the UTAUT constructs was administered to participants to elicit their experiences and challenges encountered while utilizing the app.

#### Data analysis

Feasibility metrics and the quantitative assessment of acceptability were analyzed descriptively using STATA 13. The investigator used summary statistics to analyze the sociodemographic data. For qualitative data as described in detail in Additional file 3, interviews were digitally recorded, transcribed, and analyzed using an inductive analysis approach based on the UTAUT model. In this approach, themes tailored to performance expectancy, effort expectancy, social influence, and facilitating conditions [25] were developed from the collected data. A codebook (Additional file 4) was developed by the principal investigator (WT) through categorizing themes related to performance expectancy, effort expectancy, social influence and facilitating conditions and was reviewed for meaningful data extraction by the supervisors (AM, FK, DA) through an inductive and content analysis strategy [26], which involves theme identification, elaboration and illustration of quotes. This process involves interpreting textual data content through coding and category construction. Themes and subthemes were generated to describe different data patterns and category construction about the initial acceptability and feasibility of the developed application in TB care using content analysis. The review of transcripts guided the development of a coding scheme composed of various text sections that addressed the concepts of interest, for example, the perceived ease of use, influence from friends, workmates and the requirements to use the prototype by the private healthcare workers in following up with TB patients on referral, were assigned descriptive codes. The elaboration and definitions for those illustrative codes were categorized based on particular themes. We used a qualitative data management computer software program NVIVO 11 (QSR International, Melbourne, Australia) for managing and organizing data.

#### Ethical approval

Official permission was obtained from the participating hospitals. Ethical approval was obtained from the Mbarara University Research Ethics Committee— MUREC (Protocol number: 32/03–20) and the Uganda National Council of Science and Technology— UNCST (Registration number: HS963ES). Participants provided signed informed consent before study participation.

## Results

### Sociodemographic details of participants

Twenty-two healthcare workers, including three medical doctors, fifteen nurses, three laboratory technicians and one pharmacist from three private hospitals and the MRRH TB clinic, participated in assessing the initial feasibility and acceptability of the developed mobile app. Healthcare workers were considered because they are the key users to interface with this application while patients receive SMS notifications triggered by the app, so the study capitalized on obtaining views of healthcare workers toward the application. The majority of the participants were female (*n* = 16) and were nurses (*n* = 15). Their median age was 28 years of age. All three private hospital facilities that were part of this study referred presumptive TB cases to MRRH, as indicated in Table 1.

**Table 1:** Participant demographics

Baseline characteristic	Participants
	*N (%)*
Median Age (IQR)	28 (25–29)
Gender	
Female	16 (73)
Male	6 (27)
Marital status	
Single	12 (54.5)
Married/partnered	10 (45.5)
Facility	
Public Hospital	4 (18)
Private Hospital 1	8 (36)
Private Hospital 2	5 (23)
Private Hospital 3	5 (23)
Highest educational level	
Certificate	7 (32)
Diploma	10 (45)
Degree	5 (23)
Designation	
Nurse	15 (68)
Doctor	3 (14)
Lab Technician	3 (14)
Pharmacist	1 (4)
Number of years in general medical practice	
1–5	12 (54)
	10 (46)

### The potential of Tuuka mobile application

The majority of the participants reported that the application would help them make quicker medical decisions regarding the referred presumptive patient, enhance communication with other healthcare workers about the referred patients, enhance patient follow-up and quick TB case notification, facilitate partnership between private and public hospital facilities and was perceived to be a quick and efficient referral system, as shown in Table 2 below.

**Table 2:** The potential of Tuuka mobile application among healthcare workers

Characteristic (***N*** = 22)	Agree	Strongly Agree
Helping me make quicker medical decisions	2 (9)	20 (91)
Help me communicate with other healthcare workers about the referred patient	1 (4)	21 (96)
Enhance patient follow up	1 (4)	21 (96)
Facilitate partnership with other health institutions	0	22 (100)
Quick TB case notification	1 (4)	21 (96)
Quick and efficient referral system	2 (9)	20 (91)

### Acceptability and feasibility findings

The system usability scale (SUS) [24] was used to explore how the healthcare workers perceived the usability of the application. The SUS is a 5-point Likert scale for subjective assessment of system usability. The total mean SUS score for all respondents was 98 (SD 1.97), which, according to Bangor and colleagues [27], would be in the fourth quartile range in the acceptability range. The descriptive scores of responses to the 10 questions of the SUS are presented in Table 3 below.

**Table 3:** Tuuka usability findings based on the SUS

Characteristic (***N*** = 22)	Strongly Disagree	Disagree	Agree	Strongly Agree
I think that I would like to use Tuuka application frequently	0	0	5 (23)	17 (77)
I found the Tuuka application unnecessarily complex	22 (100)	0	0	0
I thought the Tuuka application was easy to use	0	0	3	19
I think that I would need the support of a technical person to be able to use this application	18 (82)	4 (18)	0	0
I found the various functions in this application were well integrated	0	0	0	22 (100)
I thought there was too much inconsistency in this application	19 (86)	3 (14)	0	0
I would imagine that most people would learn to use this application very quickly	0	0	4 (18)	18 (82)
I found the application very cumbersome to use	21 (95)	1 (5)	0	0
I felt very confident using the application	0	0	7 (32)	15 (68)
I needed to learn a lot of things before I could get going with this application	22 (100)	0	0	0

In addition to the SUS, the UTAUT model was utilized to explore how healthcare workers perceived the acceptability of the Tuuka mobile application. Questions were structured according to the four main constructs of the UTAUT model, namely, performance expectancy, effort expectancy, social norms, and facilitating conditions. The application was found to be acceptable, and the descriptive scores of responses are presented in Table 4 below.

**Table 4:** Perceived usefulness, ease of use and social norms of the Tuuka Mobile application

Characteristic (***N*** = 22)	Neither	Agree	Strongly Agree
Tuuka mobile application is useful in following up the referred presumptive TB patients	0	7 (32)	15 (68)
Tuuka mobile application is more useful than the current referral procedures	0	6 (27)	16 (73)
Tuuka mobile application positively affected the way I feel about referring presumptive TB patients	0	5 (23)	17 (77)
Tuuka mobile application would help me in following up the referred patients in time	0	5 (23)	17 (77)
Tuuka mobile application makes it easier to follow up referred patients	1 (4)	5 (23)	16 (73)
**Perceived ease of use of Tuuka mobile app**			
It would be easy for me to use Tuuka mobile application to refer patients	0	4 (18)	18 (82)
It would easy for me to remember to use Tuuka mobile application	0	4 (18)	18 (82)
It was easy for me to install Tuuka mobile application	0	5 (23)	17 (77)
**Social norms about using Tuuka**			
People think I should use Tuuka mobile application to refer patients	0	6 (27)	16 (73)
My friends, workmates think I should use Tuuka mobile application to refer patients	0	0	22 (100)

### Facilitating conditions for Tuuka mobile application

All participants had smartphones for Tuuka application to be installed and had electricity and cellular networks as facilitating factors for the usage of the application.

### Qualitative findings

**Fig. 3:**
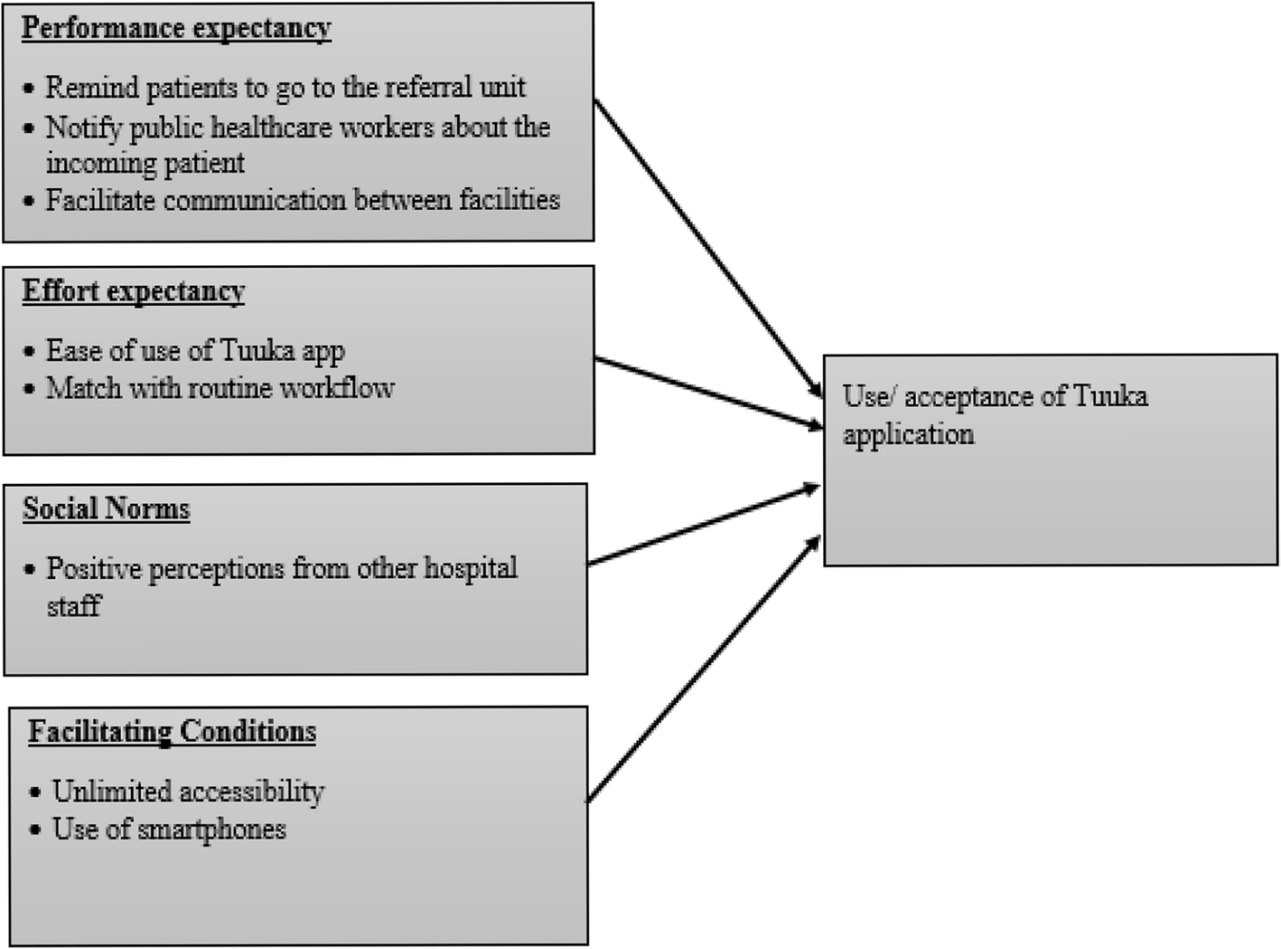
Organization of qualitative data on acceptability following the UTAUT model

The intervention acceptability results are presented following the UTAUT model (Fig. 3) and detail the performance, effort expectancy, social norms, and facilitating conditions associated with the mobile app.

### Performance expectancy

The study participants found the Tuuka mobile application useful in notifying both patients to go to the place of referral and the public healthcare workers about the referred patients. They described that the SMS notifications triggered by the Tuuka mobile application at the private health facility remind and force patients to go the place of referral and enables the healthcare worker at the public health facility to prepare to receive the referred patient.

*The application notifies the healthcare worker at the public hospital that a certain patient has been referred from a private hospital and notifies the patient to go to the place of referral. Therefore, if the patient receives this message, he or she will be forced and reminded to go to the hospital where he or she has been referred. (35-40 years old, public hospital).*

Tuuka mobile application was reported to enhance communication between facilities. The application was reported to allow healthcare workers at both private and public health facilities to communicate with each other about the referred patient after receiving an SMS notification, which would help to ascertain whether the patient reached the place of the referral or not.

*When a healthcare worker at the private hospital refers a patient, the healthcare worker at the place of referral receives a notification about the referred patient and can immediately reply in the application, which allows easy communication across facilities. (45-50 years old, private hospital 1).*

The mobile application was reported to enhance patient-centered care through coordinated patient follow-up efforts in both private and public hospitals by ensuring privacy and confidentiality of patients where they can easily be worked on without lining up since the healthcare workers are made aware in advance about the referred patient.

*This application links the referred patients by alerting the public hospitals about their coming, and they can be worked on faster by the healthcare workers upon reaching the facility. This makes a patient feel good knowing that he or she is being cared for. (25-30 years old, private hospital 2).*

### Effort expectancy

Following the healthcare workers’ initial orientation to use the application, they found it easy to use for referring presumptive TB cases. The application has an SMS functionality that would notify the healthcare worker at the public facility about the referred patient, which would prompt them to do the needful irrespective of where they are.

*I think the application does not need a lot of time to operate, it’s just a matter of adding the patients’ details and the public hospital gets notified about the referred patient. (25-30 years old, private Hospital 2).*

The participants at the referral unit noted that the application functioned well, as evidenced by receiving SMS notifications about an incoming patient as soon as the private healthcare worker referred the patient.

*The application was working very well because immediately when a patient was referred from the private hospital, I would receive the message on my phone about the incoming patient. (35-40 years old, public hospital).*

### Social influence

Participants reported positive perceptions about the intervention by other hospital staff, which increased their chances of utilizing the application.

*My fellow healthcare workers don’t have any problem with me using the app; actually, they feel it’s important in linking up the referred patients. (25-30 years old, private hospital 3).*

### Facilitating conditions

Participants noted that the application does not require much space for installation and the internet to be downloaded, which was perceived to enhance its acceptability. They reported being repulsive to downloading apps that require a lot of space and internet as it would be expensive in terms of buying internet packages in the long run.

*The application didn’t take a lot of internet bundles to download, and it doesn’t take a lot of storage space on my phone. (25-30 years old, public hospital).*

However, participants noted that the internet might limit the usability of the application since the application entirely runs on the internet. The SMS notifications sent to healthcare workers were perceived to force the healthcare worker to buy internet and obtain more information about the patient.

*You need internet so that every time you receive the SMS notification about the referred patients, you login to see the details about the referred patient. However, in case one doesn’t have internet, the SMS notification will still come, one can buy internet bundles and log in the application. (35-40 years old, public hospital).*

Participants also complained about the lack of time to utilize the application due to the busy schedules in terms of high number of patients to be worked, and at times, they would forget about the app.

*I personally would be overwhelmed with meeting patients on ward and by the time I remember using the application it would be too late. (25-30 years old, private hospital 2).*

## Discussion

In this paper, the Tuuka mobile application was reported to help healthcare workers make quicker medical decisions regarding referred patients and enhance communication with other healthcare workers and patient follow-up. Drawing from the UTAUT model, it was found that a mobile health application can be an acceptable and feasible tool for following up with referred patients from private to public hospitals among healthcare workers. The main construct to the acceptability of the application was perceived usefulness, where the developed mobile application was reported to not only have the potential to remind the referred presumptive patients to go to the place of referral but also notify healthcare workers at the public health facility about the incoming referred patient, which facilitates communication across facilities and enhances patient-based care.

Our findings reveal that notifying healthcare workers at the place of referral about the referred patients was interpreted as caring for the referred patients, which would motivate them to adhere to the referral knowing that healthcare workers at the place of referral are waiting for them. Ensuring proper notification, referral and follow-up across facilities is an important facet for continuity of care among tuberculosis patients [28] and improves care coordination [29]. Lack of these proper mechanisms for communication regarding the referred patients may compromise the continuity of treatment [30]. SMS reminders’ implication for care for patients has also been reported by [31] among people living with HIV in rural southwestern Uganda. The findings are in tandem with several literature reviews that have highlighted how SMS reminders can aid in reducing missed hospital appointments [32] and assist patients in adhering to their regimen to reduce the rates of readmission [33]. Therefore, the utilization of a mobile application that automatically sends reminders to patients and notifications to healthcare workers about the referred may enhance continuity of care among patients.

The SMS reminder component of the mobile application triggered at the private hospital may enhance timely care offered to patients, thus potentially reducing delays along the referral pathway. However, receiving a message about the referred patient among healthcare workers at the referral unit may not guarantee that the patient will be received. There could be other factors that may result in nonresponse from healthcare workers, such as a shortage of healthcare workers, which affects the delivery of healthcare services [34], and a high doctor–patient ratio. In Uganda, the doctor–patient ratio is 1:25,000 [35], which is way below the recommended WHO doctor–patient ratio of 1:1000. On the other hand, referred patients may be hindered from reaching the place of referral due to lack of transport [16], being too weak to reach the facility. Therefore, the implementers of mHealth interventions that utilize SMS should bear in mind that other factors may affect adherence to the referral even after receiving the intervention.

The application was also reported to enable communication between both private and public hospitals, which helps in keeping track of the stage where the referred patient has reached. This ensures concerted efforts from both public and private facilities to ensure that they are engaged in TB care efforts [36]. In a setting where most private hospitals are not licensed to provide TB services and where healthcare workers of these hospitals refer their presumptive patients to public health facilities [8], the application offers a follow-up mechanism for ensuring that the public hospitals are notified about each case identified in these private hospitals. Thus, enhancing PPM by garnering efforts from both actors in ensuring that the patient has been followed up to ensure that they are not lost along the referral pathway, which is key in ensuring continuity of care for patients, fosters evidence-based TB care per international standards [3].

Lack of access to the internet by healthcare workers was also noted as a potential limiting factor for the usability of the Tuuka application [22]. In settings such as Uganda, where internet penetration is still low, healthcare workers have to buy their own internet bundles to use the app, which may not be sustainable in the long run. The study findings have shown that mobile apps that require more internet bundles to be downloaded and consume a lot of space on the users’ devices are likely not to be utilized. Users are likely to abandon mobile applications that take up considerable space or memory on their phones [37]. Therefore, developers should take this into consideration.

There were no challenges with user motivation reported in this study, probably due to the limited amount of time the intervention was used. However, the busy working nature of healthcare workers coupled with the prevailing high patient–doctor ratio may constrain the use of the app. Most mobile health interventions lack sustained use and are later abandoned by the intended users mainly due to lack of motivation or declining interest in using the intended intervention [38]. Future studies can explore the possible ways of motivating staff (especially at private facilities) to use the app.

### Implications for policy and implementation

This study implies that a mobile application for following up presumptive TB patients referred from private to public hospitals is acceptable and feasible. However, the development of mobile health interventions for healthcare workers should be done with caution due to the nature of their work [22]. Therefore, the integration of these interventions in the routine work of healthcare workers needs to be carefully planned if the required results are to be achieved. Second, the underlying technological development used to develop the application can be expanded in following up all patients referred from private to public hospitals. The application enables the automation of the referral process, especially in settings where public–private referrals are common, and offers valuable insights and knowledge necessary for the development of a similar intervention in other settings.

### Limitations of the study

A purposive sampling approach for recruiting the respondents was utilized, which has the potential for selection bias. In addition, although this study recruited all healthcare workers who met the selection criteria (experience in TB-related case identification, referral and treatment), the sample size was small, which limits the ability to generalize the study findings among healthcare workers in similar settings. This study was conducted in an urban setting; these findings may not be generalizable in other settings, such as rural areas, where there might be considerable differences in implementation and practices. Importantly, this study did not assess patients’ perceptions of the app. This was because the key users to interface (install) with this application are healthcare workers, while patients receive SMS notifications triggered by the app, so the study capitalized on obtaining views of healthcare workers toward the application. Although our study was based on healthcare workers (doctors, nurses and laboratory technicians) to test the developed Tuuka application since they are its main users, involving external experts to walk through the solution and provide their opinion would have increased the confidence in the developed solution. To the best of our knowledge, our study utilized a user-centered approach that involves system users in intervention development, testing and refinement, and including system experts in future studies by developers can enhance the development of systems that can be trusted.

## Conclusion

This study showed that the Tuuka mHealth application for private hospitals to notify public hospitals about the referred presumptive TB patients is acceptable and feasible for following up with the referred presumptive TB patients referred from private to public hospitals in southwestern Uganda. The key aspects of intervention acceptability and feasibility were performance expectancy in terms of notifying healthcare workers at the public hospital about the incoming referred patient and facilitating communication across facilities, thus enhancing patient-based care. Future efforts can explore larger studies with a longer-term follow-up for assessing clinical outcomes among patients and healthcare workers.

## Supplementary Information

**Additional file 1.** A questionnaire for assessing the acceptability and feasibility of the Tuuka mobile application among health care workers.

**Additional file 2.** An interview guide for assessing acceptability and feasibility.

**Additional file 3.** COREQ Checklist for detailing qualitative data.

**Additional file 4.** A codebook detailing the code names, description and illustrative quotes regarding the acceptability and feasibility of the Tuuka Mobile application.

## Data Availability

The datasets used and/or analyzed during the current study are available from the corresponding author on reasonable request.
