# Supplementary material for: Acceptability and feasibility of a mobile health application for enhancing public private mix for TB care among healthcare Workers in Southwestern Uganda
Source: BMC Digit Health. 2023 Mar 3;1(1):9. doi: 10.1186/s44247-023-00009-0 (PMC9982777; doi:10.1186/s44247-023-00009-0)
Supplement: Supplementary file 1 — Additional file 1. A questionnaire for assessing the acceptability and feasibility of the Tuuka mobile application among health care workers. [file 44247_2023_9_MOESM1_ESM.doc]

**A Questionnaire for assessing the acceptability and feasibility of Tuuka mobile application among Health Care workers.**

**Study title:** A Mobile health Framework for Public Private Mix in Tuberculosis prevention and Care.

**Study Objective:** To explore the feasibility and acceptability of a mobile intervention for following up referred presumptive TB patients from private to public hospitals rural South Western Uganda.

My name is Wilson Tumuhimbise, a PhD student of Mbarara University of Science and Technology, REG NO, 2018/PhD/030/PS. I am carrying out a study entitled “A Mobile health Framework for Public Private Mix in Tuberculosis prevention and Care”. Mobile health (mHealth) is defined as the use of mobile and wireless technologies to achieve health objectives and this study is about exploring the acceptability and feasibility of a mobile intervention for following up referred presumptive TB patients from private to public hospitals. Participation is voluntary, filling this questionnaire will only take you 35 to 40 minutes. The response you give will be treated with utmost privacy.

**Circle the Alternative of your choice.**

| **PART A: SOCIAL DEMOGRAPHIC DATA** | |
| --- | --- |
| **Participant No…….. *(****For the researcher****)*** | |
| 1. Date of Birth |___|___|___| | 1. Gender **MALE**  **FEMALE** |
| 1. Marital Status: **Married**  **Single** | 1. Name of the Health Facility: **………………………..** |
| 1. Type of the Facility: Private for Profit  Faith based  Private Not for Profit | 1. Highest level of education achieved:   Certificate  Diploma  Degree  Masters  PhD |
| 1. Designation: Nurse  Doctor  Lab Technician  Pharmacist  Other ………………………………….. | 1. Number of years of practice   <1  1-3 4- 5  >5 |

**The potential of Tuuka mobile application among healthcare workers**

| 1= **Strongly Disagree 2= Disagree, 3= Not Sure, 4= Agree, 5= Strongly Agree** | **1** | **2** | **3** | **4** | **5** |
| --- | --- | --- | --- | --- | --- |
| Helping me make quicker medical decisions |  |  |  |  |  |
| Help me communicate with other health care workers about TB disease |  |  |  |  |  |
| Enhance patient follow up |  |  |  |  |  |
| Facilitate partnership with other health institutions |  |  |  |  |  |
| Quick TB case notification |  |  |  |  |  |
| Quick and efficient referral system |  |  |  |  |  |

| **The System Usability Scale for assessing acceptability at the end of using the intervention** | | | | | | | | |
| --- | --- | --- | --- | --- | --- | --- | --- | --- |
|  |  |  | **Strongly Disagree** | | | **Strongly agree** | | |
| 1 | I think that I would like to use Tuuka app frequently |  |  |  |  | |  |  |
|  |  |  | 1 | 2 | 3 | | 4 | 5 |
| 2 | I found the Tuuka app unnecessarily complex |  |  |  |  | |  |  |
|  |  |  | 1 | 2 | 3 | | 4 | 5 |
| 3 | I thought the Tuuka app was easy to use |  |  |  |  | |  |  |
|  |  |  | 1 | 2 | 3 | | 4 | 5 |
| 4 | I think that I would need the support of a technical person to be able to use this app |  |  |  |  | |  |  |
|  |  |  | 1 | 2 | 3 | | 4 | 5 |
| 5 | I found the various functions in this app were well integrated |  |  |  |  | |  |  |
|  |  |  | 1 | 2 | 3 | | 4 | 5 |
| 6 | I thought there was too much inconsistency in this app |  |  |  |  | |  |  |
|  |  |  | 1 | 2 | 3 | | 4 | 5 |
| 7 | I would imagine that most people would learn to use this app very quickly |  |  |  |  | |  |  |
|  |  |  | 1 | 2 | 3 | | 4 | 5 |
| 8 | I found the app very  cumbersome to use |  |  |  |  | |  |  |
|  |  |  | 1 | 2 | 3 | | 4 | 5 |
| 9 | felt very confident using the app |  |  |  |  | |  |  |
|  |  |  | 1 | 2 | 3 | | 4 | 5 |
| 10 | I needed to learn a lot of  things before I could get going with this app |  |  |  |  | |  |  |
|  |  |  | 1 | 2 | 3 | | 4 | 5 |

**Quantitative Survey for Technology Adoption**

Hope you are fine. You have been participating in a study that utilized the Tuuka mobile application for following up the referred presumptive TB patients referred from private to public hospitals. The following questions are meant to help us understand your experience of using the intervention.

**Perceived Usefulness of the Tuuka Mobile application**

1. Using the Tuuka mobile app is useful in following up the referred presumptive TB patients from private to public hospitals.

❑Strongly Agree ❑Agree ❑Disagree ❑Strongly Disagree

1. Using the Tuuka mobile app is more useful in following up the referred presumptive TB patients from private to public hospitals than the current referral procedures. ❑Strongly Agree ❑Agree ❑Disagree ❑Strongly Disagree.
2. Using the Tuuka mobile app positively affected the way I feel about referring presumptive TB medicine. ❑Strongly Agree ❑Agree ❑Disagree ❑Strongly Disagree.
3. Using Tuuka mobile app helped me followup the referred presumptive TB patients in time/as prescribed. ❑Strongly Agree ❑Agree ❑Disagree ❑Strongly Disagree.
4. Using Tuuka mobile app makes it easier to follow up presumptive TB patients referred from private to public hospitals.

❑Strongly Agree ❑Agree ❑Disagree ❑Strongly Disagree.

**Perceived Ease of Use of the Tuuka mobile app**

1. It was easy for me to use Tuuka mobile app to refer presumptive TB patients to public hospital.

❑Strongly Agree ❑Agree ❑Disagree ❑Strongly Disagree.

1. It was easy for me to remember how to use Tuuka mobile app.

❑Strongly Agree ❑Agree ❑Disagree ❑Strongly Disagree.

1. It was easy for me to install Tuuka mobile app on my phone. ❑Strongly Agree ❑Agree ❑Disagree ❑Strongly Disagree.

**Social Norms about using Tuuka application**

1. People who take care of my health think I should use Tuuka mobile app for following up presumptive TB patients referred from private to public hospitals ❑Strongly Agree ❑Agree ❑Disagree ❑Strongly Disagree.
2. People who influence my behaviour think I should use the wisepill device Tuuka mobile app for following up presumptive TB patients referred from private to public hospitals. ❑Strongly Agree ❑Agree ❑Disagree ❑Strongly Disagree.
3. People who are important to me think I should use Tuuka mobile app for following up presumptive TB patients referred from private to public hospitals. ❑Strongly Agree ❑Agree ❑Disagree ❑Strongly Disagree.

**Facilitating Conditions**:

1. I possess a personal smart phone needed to install Tuuka mobile application. ❑Strongly Agree ❑Agree ❑Disagree ❑Strongly Disagree.
2. My personal smartphone allows Tuuka mobile app to successful operate. ❑Strongly Agree ❑Agree ❑Disagree ❑Strongly Disagree.
3. I have electricity/solar for charging my mobile phone. ❑Strongly Agree ❑Agree ❑Disagree ❑Strongly Disagree.
4. I have reliable mobile telephone network at the hospital facility that could enable me to use Tuuka. ❑Strongly Agree ❑Agree ❑Disagree ❑Strongly Disagree.
5. I used Tuuka mobile app consistently. ❑Strongly Agree ❑Agree ❑Disagree

❑Strongly Disagree.

*✿****Thank you for your help****✿*
