# Supplementary material for: Acceptability and feasibility of a mobile health application for enhancing public private mix for TB care among healthcare Workers in Southwestern Uganda
Source: BMC Digit Health. 2023 Mar 3;1(1):9. doi: 10.1186/s44247-023-00009-0 (PMC9982777; doi:10.1186/s44247-023-00009-0)
Supplement: Supplementary file 2 — Additional file 2. An interview guide for assessing acceptability and feasibility. [file 44247_2023_9_MOESM2_ESM.docx]

**INTERVIEW GUIDE for assessing acceptability and feasibility**

1. How are you doing today?
2. Tell me about your experience with using a mobile phone based application for tracking TB patients referred from private to public hospital? Probe More Is it easy or difficult to use, is it flexible? Can you use it any time you want?
3. Tell me about your experience with using a mobile phone based application for TB care that you received. (Referral, notification and identification, linking to different health Centers).
4. In what ways was it easy/hard to learn to use the mobile phone based application?
5. How did it help you in caring for TB patients? What do you like/ dislike about it?
6. What challenges have you encountered in using this application? (probe for: app failure, network connectivity, convenience)
7. How do you feel about the usage of mobile health for tracking TB patients referred from private to public hospitals?
8. How do you compare the use of a mobile phone based app for tracking TB patients referred with other traditional means (e.g. Referral notes)
9. What do others (family members, friends, and people around you) think about your usage of mobile health for tracking TB patients referred from private to public hospital? Do they understand it? What is their reaction when they see you use that application for TB disease management?
10. Any questions for me?
11. Thank you for your response and thank you for being part of the study and interview.
