# Supplementary material for: Acceptability and feasibility of a mobile health application for enhancing public private mix for TB care among healthcare Workers in Southwestern Uganda
Source: BMC Digit Health. 2023 Mar 3;1(1):9. doi: 10.1186/s44247-023-00009-0 (PMC9982777; doi:10.1186/s44247-023-00009-0)
Supplement: Supplementary file 3 — Additional file 3. COREQ Checklist for detailing qualitative data. [file 44247_2023_9_MOESM3_ESM.docx]

**COREQ Checklist**

| **Domain 1:**   \| **Research team and reflexivity** \| \| --- \| |  | \| Location in manuscript (Section, page no.) \| \| --- \| |
| --- | --- | --- | --- | --- |
| **Personal characteristics** |  |  |
| 1. Interviewer/facilitator: Which author/s conducted the interview or focus group? | **WT** | **Methods-9** |
| 1. **Credentials:** What were the researcher’s credentials? E.g. PhD, MD | **MSc, BIT** | **-** |
| 1. What was their occupation at the time of the study? | **Principal Investigator** | **-** |
| 1. **Gender:** Was the researcher male or female? | **Male** | **-** |
| 1. Experience and training: What experience or training did the researcher have? | At the time of the interviews, the researcher had completed Responsible conduct of Research training where qualitative research methods were covered at length | **-** |
| **Relationship with participants** |  |  |
| 1. Relationship established: Was a relationship established prior to study commencement? | **Yes** | **-** |
| 1. Participant knowledge of the   Interviewer: What did the participants know about the researcher? e.g. personal goals, reasons for doing the  research | All participants were briefed on the purpose of the study and understood that it was a research project aimed at exploring the feasibility and acceptability of a mobile application for enhancing public private mix for Tuberculosis care. They were informed at enrollment about their right to refuse/withdraw from the study at any time without any penalty or losing the benefits they were entitled to at the hospital facility.  They provided signed informed consent before study participation | **Ethical approval-11-12** |
| 1. Interviewer characteristics: What characteristics were reported about the interviewer/facilitator? e.g. Bias, assumptions, reasons and interests in the research topic | No interviewer-related biases identified | **-** |
| **Domain 2: study design** |  |  |
| Theoretical framework |  |  |
| 1. Methodological orientation and Theory: What methodological orientation was stated to underpin the study? e.g. grounded theory,   discourse analysis, ethnography, phenomenology, content analysis | Inductive content analytic approach | **Methods: Page 11** |
| **Participant selection** |  |  |
| 1. Sampling: How were participants selected? e.g. purposive, convenience, consecutive, snowball | We purposively selected with purposefully selected healthcare workers from three private hospitals that referred patients to Mbarara Regional Referral Hospital TB clinic to understand how feasible and acceptable an mHealth intervention for enhancing public private mix for TB care is. | **Methods: Page 5** |
| 1. Method of approach: How were participants approached? e.g. face-to-face, telephone, mail, email | telephone | **Methods-6** |
| 1. Sample size: How many participants were in the study? | 22 | **Methods-10** |
| 1. Non-participation: How many people refused to participate or dropped out? Reasons? | Two of the participants reported failure to download the application from the shared link and were not available to participate in data collection. | **Methods-10** |
| **Setting** |  |  |
| 1. Setting of data collection: Where was the data collected? e.g. home, clinic, workplace | Data was collected via telephone | **Methods -6** |
| 1. Presence of non-participants: Was anyone else present besides the participants and researchers? | No |  |
| 1. Description of sample: What are the important characteristics of the sample? e.g. demographic data, date | Median age=28, 73% were female. | **Table 1** |
| **Data collection** |  |  |
| 1. Interview guide: Were questions, prompts, guides provided by the authors? Was it pilot tested? | Yes | **Methods- 10** |
| 1. Repeat interviews: Were repeat interviews carried out? If yes, how many? | No |  |
| 1. Audio/visual recording: Did the research use audio or visual recording to collect the data? | Yes. Interviews were digitally recorded, transcribed, and translated to English | **Methods- 11** |
| 1. Field notes: Were field notes made during and/or after the interview or focus group? | No additional field notes were made |  |
| 1. Duration: What was the duration of the interviews or focus group? | Each interview lasted between 30 and 45 minutes | **Methods- 10** |
| 1. Data saturation: Was data saturation discussed? | Interviews were carried out until thematic saturation was reached at the 22^nd^ participant and there was no new themes coming out from the data except repetition | **Methods-7** |
| 1. Transcripts returned: Were transcripts returned to participants for comment and/or correction? | No |  |
| **Domain 3: analysis and findings** |  |  |
| Data analysis |  |  |
| 1. Number of data coders: How many data coders coded the data? | One | **Methods 11** |
| 1. Description of the coding tree: Did authors provide a description of the coding tree? | Inductive content analysis. (Theme identification, elaboration and illustration of quotes) | **Methods 11** |
| 1. Derivation of themes: Were themes identified in advance or derived from the data? | Derived from the data | **Methods-11** |
| 1. Software: What software, if applicable, was used to manage the data? | N/A |  |
| 1. Participant checking: Did participants provide feedback on the findings? | No |  |
| Reporting |  |  |
| 1. Quotations presented: Were participant quotations presented to illustrate the themes / findings? Was each quotation identified? e.g. participant number | Yes, specific themes were supported with illustrative quotes attributed to anonymised participant details. | **Results 15-18** |
| 1. Data and findings consistent: Was there consistency between the data presented and the findings? | Yes |  |
| 1. Clarity of major themes: Were major themes clearly presented in the findings? | Yes |  |
| 1. Clarity of minor themes: Is there a description of diverse cases or discussion of minor themes? | Minor themes are discussed in the manuscript |  |
