# Supplementary material for: Acceptability and feasibility of a mobile health application for enhancing public private mix for TB care among healthcare Workers in Southwestern Uganda
Source: BMC Digit Health. 2023 Mar 3;1(1):9. doi: 10.1186/s44247-023-00009-0 (PMC9982777; doi:10.1186/s44247-023-00009-0)
Supplement: Supplementary file 4 — Additional file 4. A codebook detailing the code names, description and illustrative quotes regarding the acceptability and feasibility of the Tuuka Mobile application. [file 44247_2023_9_MOESM4_ESM.docx]

| Code Name | Code description and properties | Examples quote(s) |
| --- | --- | --- |
| Performance Expectancy or perceived ease of use | The potential of Tuuka app to notify both patients to go to the place of referral and the public healthcare workers about the referred patient | *R: The application* *notifies the healthcare worker at the public hospital that a certain patient has been referred from a private hospital and also notifies the patient to go to the place of referral. So if the patient receives this message, he or she will be forced and reminded to go to the hospital where he has been referred. (35-40 years old, public hospital).*  *R: The application prepares the healthcare worker to be alert knowing that there is a patient coming from the private hospital and also enables the healthcare worker to get more information about the history of the incoming patient which enables the healthcare worker to prepare enough and to know what to do when the patient comes to the facility and it forces the healthcare worker to follow up the patient and know whether this patient reached the hospital. (35-40 years, Public Hospital)* |
|  | The potential of the app to enhance patient centered care through coordinated patient follow up efforts in both the private and public health facilities | *R: Usually patients from private hospital always require privacy and confidentiality, they don’t want to be in those long lines, so this app helps in connecting them by alerting the public health facility about their coming and they can be worked on faster by the healthcare workers upon reaching the facility. This makes a patient feel good knowing that he or she is being cared for. (25-30 years old, private hospital 3).*  *R: This application links the referred patients by alerting the public hospitals about their coming and they can be worked on faster by the healthcare workers upon reaching the facility. This makes a patient feel good knowing that he or she is being cared for. (25-30 years old, private hospital 2).* |
|  | The potential of Tuuka to enhance communication between facilities | *R: If every facility can have that application so that when they refer the patient and notify cases through the application it becomes an easiest way to communicate with other facilities to follow up. Because when the person at the private hospital refers a patient, you receive a message that a patient has been referred, so when you receive that patient, you also immediately reply in the application which brings that easy communication across facilities. (30-45 years old, private hospital 1).*  *R: When a healthcare worker at the private hospital refers a patient, and healthcare worker at the place of referral receives a notification about the referred patient and can immediately reply in the application which brings that easy communication across facilities. (45-50 years old, private hospital)* |
| Effort expectancy or perceived ease of use | The potential of Tuuka mobile app to be easy to use for referring the presumptive TB cases | *R: I think the app does not need a lot of time to operate, it’s just a matter of adding the patients’ details and the public hospital gets notified about the referred patient. The application was working very well because immediately when a patient was referred from the private hospital, I would receive the message on my phone about the incoming patient. (Female, Nurse, 25 years, Private Hospital).*  *R: The application was working very well because immediately when a patient was referred from the private hospital, I would receive the message on my phone about the incoming patient. (35-40 years old, public hospital).*  *R: The interfaces were fast and easy, it is user friendly, you just put it details and it is simple it directs you to the next stage. The app is not so robust therefore acceptability on the users will be faster because people don’t like a lot of data entry, so when it has few data entries, it makes the process simpler. (Male, Lab technician, 46 years, Private Hospital)* |
| Social influence | Positive perception about the intervention | R: *My fellow healthcare workers don’t have any problem with me using the app, actually they feel it’s important in linking up the referred patients. (25-30 years old, private hospital 3).* |
| Facilitating Conditions | Facilitating conditions for using Tuuka mobile application | *The application didn’t take a lot of internet bundles to download, and, it doesn’t take a lot of storage space on my phone. (25-30 years old, public hospital).*  *You need internet so that every time you receive the SMS notification about the referred patients, you login to see the details about the referred patient. But in case one doesn’t have internet, the SMS notification will still come, one can buy internet bundles and log in the application. (35-40 years old, public hospital).*  *I personally would be overwhelmed with meeting patients on ward and by the time I remember using the application it would be too late. (25-30 years old, private hospital 2).* |
